# Relative and absolute blood flow values during cardiopulmonary bypass

**DOI:** 10.1051/ject/2023008

**Published:** 2023-06-28

**Authors:** Ignazio Condello

**Affiliations:** 1 Department of Cardiac Surgery, Anthea Hospital, GVM Care & Research, Perfusion Service Via Camillo Rosalba 35/37 70124 Bari Italy

Blood flow (BF) management during cardiopulmonary bypass (CPB) is crucial for preventing injury to vital organs. The quantitative aspects of BF have been associated with cardiac index and body surface area and they are managed according to metabolic parameters, such as the relationship between delivery and oxygen consumption in relation to temperature, and hemodynamic parameters, such as mean arterial pressure. The qualitative aspects of BF are managed via different types of pumps (roller or centrifugal) with either a pulsed or continuous flow. However, the quantity and quality of BF measured during CPB weaning remain relative and not absolute specifying parameters, especially during the establishment and weaning phases of CPB, owing to the use of vents in the aortic root that sequesters BF or replenish bleeding via aspiration, which constitutes a theft of systemic flow. In such circumstances, the reintegration of the value stolen by endo-cavitary aspiration or bleeding (considered as a dynamic shunt to the circulation) is advisable.

In this context, we read with great interest the article titled “Pulsatile versus nonpulsatile blood flow during cardiopulmonary bypass” by Chaney. In this interesting review, the author highlights the importance and advantages of pulsatile flow compared with non-pulsatile flow. Multiple studies that have evaluated the efficacy of pulsatile flow during CPB have reported controversial results [1].

The suggested benefits of pulsatile perfusion include reduced systemic inflammatory response syndrome associated with bypass, decreased need for inotropic support, shortened hospital stay, and superior organ preservation [2]. We send this letter to emphasize the concept of relative and absolute flow in pulsatile management, which appears to have been taken for granted in the calculations used by modern heart-lung machines. In future studies, it would be interesting to evaluate the role of relative and absolute BF during the establishment and weaning phases of CPB on the pulsatility index and correlate the same with improvements in clinical outcomes.
